# Colorectal cancer screening challenges in southwestern Saudi Arabia

**DOI:** 10.1097/MD.0000000000049289

**Published:** 2026-06-12

**Authors:** Abdullah A. Al-Shahrani, Omair M. Alshahrani, Dima M. Alshahrani, Mohammed A. Ogran, Faisal M. AlShehry, Saifaleslam A. Mahmoud, Yara A. Alshehri, Yahya Shabi, Abdulrahman Alloghbi, Ibrahim H. Tawhari, Mohammed A. Alzahrani

**Affiliations:** a Department of Medicine, College of Medicine, King Khalid University, Abha, Saudi Arabia; b General Medicine Practice Program, Batterjee Medical College, Aseer, Saudi Arabia; c Department of Microbiology and Clinical Parasitology, College of Medicine, King Khalid University, Abha, Saudi Arabia.

**Keywords:** colon cancer, colonoscopy, colorectal cancer, fecal immunochemical testing, rectal cancer, screening

## Abstract

Colorectal cancer (CRC) is the third most common malignancy, but ranks second in cancer-related mortality. CRC screening has been shown to decrease the incidence of CRC. However, screening methods are only effective if they are done. This is an analytical cross-sectional study done via a web-based questionnaire that was distributed in person and online. Convenient sampling methods were used to collect responses from adult males and females willing to participate in this study. The study included 643 participants with female predominance (72.2%). Although 88.5% recognized CRC as a preventable disease and 68.4% knew early detection improves outcomes, only 35.1% were aware of cancer screening tests. The most reported barriers to general screening were absence of symptoms (63.3%) and lack of physician recommendation (57.5%). Emotional and psychological barriers were the most reported barriers for colonoscopy (62.5%), and lack of knowledge about the availability of stool-based tests (50.5%). The study participants demonstrated fair knowledge about risk factors and symptoms of CRC, but there were significant knowledge gaps about the available screening methods. This highlights the need for national awareness campaigns and improved patient-physician communication about CRC screening.

## 1. Introduction

Colorectal cancer (CRC) is the third most commonly diagnosed cancer worldwide and ranks second in cancer-related mortality.^[1]^ In 2022, about 1.9 million new cases of CRC were reported, resulting in approximately 935,000 deaths.^[2]^ The epidemiology of CRC varies globally with around a 10-fold variation in colon cancer incidence by region.^[2]^ The highest incidence of colon cancer was reported in Europe and Australia/New Zealand, while it was low in Africa and south/central Asia.^[2]^ Generally, developed countries have higher rates of CRC than developing countries. However, a trend towards higher rates has been observed in countries undergoing major transitions into industrialization. This increase was attributed in part to lifestyle and dietary changes (increased consumption of animal-based proteins and processed foods), leading to higher rates of obesity.^[1,2]^ In Saudi Arabia, CRC incidence was estimated to be 14.6%, with gender variation as males had an incidence rate of 19.6% while females had a 9.5% rate.^[3]^ Between 2006 and 2016, the number of colon cancer cases in Saudi Arabia increased by 8%, while rectal cancer increased by 7% over the same time frame.^[4]^ The Middle East and North Africa region witnessed an increase in age-standardized incidence rate by 40% between 1990 and 2017, while in Saudi Arabia, it increased by 150% during the same time frame.^[5]^ The mortality rate of CRC among the Saudi population was 1.48%.^[3,4]^

The incidence of CRC among developed countries has declined over the past decade, which was thought to be related to effective screening programs as well as diet and lifestyle modifications.^[6,7]^ The increased uptake of screening colonoscopy and subsequent removal of precursor lesions has contributed to the decline in incidence in once high-incidence countries.^[8–10]^ Colorectal screening programs have been shown to be cost-effective in countries with limited resources as well.^[11,12]^ Colorectal screening has been recommended to asymptomatic, average-risk people starting at age 45.^[13]^ CRC screening uptake depends on multiple factors, but one of the most important ones would be awareness about such programs. A study in western Saudi Arabia utilizing a questionnaire showed that only 37.4% of the participants had heard about CRC screening programs.^[14]^ Another study in central Saudi Arabia showed that 42.9% of participants believed that colon cancer screening should be initiated upon symptom onset, while a significant proportion of them did not appreciate risk factors for CRC.^[15]^ A more recent study done in Eastern Saudi Arabia showed that only 10% of study participants were informed about the CRC screening program, and about 10% had undergone a screening colonoscopy.^[16]^ Only about 22.1% of study participants in the Asir region knew the appropriate timing of CRC screening.^[17]^

Given the inadequate knowledge level about CRC and the effects it bears on screening among the population in Saudi Arabia in general, and those living in the Asir region, specifically. We aim to investigate the knowledge and perceived barriers to CRC screening. We hope to identify knowledge gaps, misconceptions, and attempt to devise a plan to correct them to improve CRC screening uptake.

## 2. Materials and methods

This is a cross-sectional study utilizing a web-based questionnaire that was distributed in person (malls, primary health care centers, public spaces) and online (through social media platforms “WhatsApp”). A convenient sampling method was utilized to collect responses from adult (>18 years of age) males and females who were willing to participate in and complete the questionnaire between October 1, 2024, and February 4, 2025. Exclusion criteria: children (<18 years of age), those with visual impairment who cannot undertake the questionnaire, and those who do not complete or consent to the questionnaire. The required sample size was determined using the single-proportion formula, with a 95% confidence level, a 5% margin of error, and an assumed prevalence of 50% for awareness or uptake of CRC screening. The choice of 50% reflects the conservative assumption that maximizes variability and thus provides the largest required sample in the absence of precise local estimates. This calculation yielded a minimum of 384 participants, which was rounded to 385 to account for potential nonresponse and to ensure adequate precision for the study objectives. The questionnaire contained multiple domains, including demographics, knowledge about the colon and CRC, and perceived challenges to CRC screening. This was compiled after a comprehensive review of pertinent literature and current guidelines. The initial draft was reviewed by 2 gastroenterologists and an oncologist for content validity. Each item was assessed for clarity, relevance, and suitability. Face validity was appraised during the pilot testing involving 60 participants to ascertain comprehensibility and completion. Internal consistency was assessed using the Kuder-Richardson Formula 20 (KR20) for binary knowledge questions and Cronbach alpha for Likert scale questions. The questionnaire demonstrated good internal consistency across all main subscales with risk factors and symptoms knowledge subscales yielding KR-20 coefficients of 0.820 and 0.807, respectively. The combined barriers subscale yielded a Cronbach alpha of 0.868.

Considering that the survey is completely voluntary and anonymous, participation in the survey was considered implied consent. Ethical approval was sought and obtained from the Research Ethics Committee at King Khalid University (ECM#2024-1303). The collected data were stored in an encrypted and password-protected drive. The data collected were designed to be anonymous; therefore, no patient identifiers were collected.

### 2.1. Statistical analysis

Descriptive statistics were calculated to characterize the demographic profile of the 643 participants, presenting frequencies and percentages for categorical variables. All statistical analyses were conducted using IBM SPSS version 29.0.0.0.

## 3. Results

The study included 643 participants, with a predominant female representation (n = 464, 72.2%) compared to males (n = 179, 27.8%). Age distribution showed that most participants were in the 18 to 29 years age group (n = 215, 33.4%). The demographic characteristics of the participants are presented in Table 1.

**Table 1:** Demographic characteristics.

Variable	N (%)
Sex	
Male	179 (27.8%)
Female	464 (72.2%)
Age	
18–29	215 (33.4%)
30–39	95 (14.8%)
40–49	169 (26.3%)
50–59	114 (17.7%)
60–69	39 (6.1%)
70 and above	11 (1.7%)
Education level	
Secondary education and below	179 (27.8%)
Bachelors/diploma	425 (66.1%)
Postgraduate	39 (6.1%)
Profession	
Unemployed	352 (54.7%)
Government employee	214 (33.3%)
Private sector	55 (8.6%)
Health care worker	22 (3.4%)
Marital status	
Single	223 (34.7%)
Married	390 (60.7%)
Divorced/widowed	30 (4.7%)
Do you have first-degree relatives with colon cancer?	
Yes	44 (6.8%)
No	599 (93.2%)

### 3.1. General knowledge, knowledge of risk factors, symptoms, and screening

The majority of participants (n = 487, 75.7%) had prior awareness of CRC. A significant proportion (n = 569, 88.5%) recognized that early detection improves treatment outcomes, and 68.4% (n = 440) acknowledged it as a preventable disease. However, only 35.1% (n = 226) were aware of cancer detection tests. Nearly half of the participants (n = 319, 49.6%) believed CRC was common in Saudi Arabia. Most participants demonstrated positive attitudes toward screening, with 67.3% (n = 433) disagreeing that screening was useless, and 59.7% (n = 384) recognizing CRC as a serious health threat. Knowledge of risk factors was generally good, with family history being the most widely recognized risk factor (n = 471, 73.3%), followed by smoking (n = 464, 72.2%) and inflammatory bowel diseases (n = 459, 71.4%). Participants demonstrated good awareness of common symptoms, with unintentional weight loss being the most recognized symptom (n = 470, 73.1%), followed by blood in stool or dark stool (n = 447, 69.5%), and sudden changes in bowel habits in the elderly (n = 445, 69.2%; Material S1, Supplemental Digital Content).

Interestingly, a substantial proportion (27.2%, n = 175) did not know the anatomical location of the colon, while smaller percentages incorrectly identified the colon as part of the small intestine (10.7%, n = 69) or stomach (5.4%, n = 35). Knowledge about the recommended screening age showed considerable variation. The most common response was 41 to 50 years (25.5%, n = 164), followed by those who indicated they did not know (20.2%, n = 130). Alarmingly, 17.7% (n = 114) incorrectly believed screening should only begin when symptoms appear. Most participants (76.5%, n = 492) reported no family history of CRC, while 16.0% (n = 103) did. A small percentage (7.5%, n = 48) were uncertain about their family history. About half of the participants (52.3%, n = 336) expressed satisfaction with CRC screening practices, while a smaller proportion (17.0%, n = 109) were dissatisfied (Table 2).

**Table 2:** Basic knowledge, screening awareness, and attitudes towards colorectal cancer.

Response	N (%)
The colon is considered one of	
Small intestine	69 (10.7%)
Stomach	35 (5.4%)
Large intestine	364 (56.6%)
I do not know	175 (27.2%)
The recommended age to start early screening for colon cancer for the general public is	
21–30	89 (13.8%)
31–40	83 (12.9%)
41–50	164 (25.5%)
51–60	54 (8.4%)
61–70	9 (1.4%)
Only when symptoms are present	114 (17.7%)
I do not know	130 (20.2%)
Are you keen to attend awareness seminars about colon cancer?	
Yes	194 (30.2%)
No	360 (56.0%)
Neutral	89 (13.8%)
Do you have a family history of colon cancer?	
Yes	103 (16.0%)
No	492 (76.5%)
I do not know	48 (7.5%)
How satisfied are you with early screening for colon cancer among the general public?	
Satisfied	336 (52.3%)
Neutral	198 (30.8%)
Not satisfied	109 (17.0%)

### 3.2. General screening barriers

The most prominent reported barriers to general screening were the absence of symptoms (n = 407, 63.3%) and lack of physician recommendation (n = 370, 57.5%). A significant knowledge gap was evident as 79.8% (n = 513) were unaware of available screening types. Although most participants recognized CRC as a serious health threat (n = 441, 68.6%) and believed in colonoscopy’s effectiveness (n = 394, 61.3%), almost half of the participants (42.3%, n = 272) cited the nonmandatory nature of screening as a barrier.

### 3.3. Colonoscopy-specific barriers

Emotional and psychological barriers were predominant for colonoscopy screening. Embarrassment was the most reported barrier (n = 402, 62.5%), followed by fear of results (n = 297, 46.2%), and perception of pain (n = 281, 43.7%). Procedural concerns included uncertainty about eligibility (n = 260, 40.4%) and time consumption (n = 251, 39.0%). Financial barriers were noted by 36.1% (n = 232) who considered colonoscopy expensive. Notably, most participants (n = 416, 64.7%) had not experienced previous negative experiences with colonoscopy, and 54.0% (n = 347) recognized its importance.

### 3.4. Fecal immunochemical test barriers

The primary barrier to fecal immunochemical test (FIT) was lack of knowledge about test availability (n = 325, 50.5%), followed by fear of results (n = 271, 42.1%), and discomfort with the testing procedure (n = 244, 37.9%). Time constraints were reported by 30.2% (n = 194) of participants. However, most participants recognized the importance of FIT (n = 335, 52.1% disagreed that it was unimportant), and cost was not perceived as a major barrier, with only 17.0% (n = 109) considering it expensive (Table 3).

**Table 3:** Barriers to colorectal cancer screening.

Statement	Agree (N, %)	Neutral (N, %)	Disagree (N, %)
Barriers to colon cancer screening			
Colon cancer screening is not mandatory.	272 (42.3%)	211 (32.8%)	160 (24.9%)
I think colonoscopy is ineffective.	80 (12.4%)	169 (26.3%)	394 (61.3%)
Colon cancer is not a serious health threat.	83 (12.9%)	119 (18.5%)	441 (68.6%)
I do not have transportation to come for the examination.	130 (20.2%)	167 (26.0%)	346 (53.8%)
No doctor’s recommendation.	370 (57.5%)	139 (21.6%)	134 (20.8%)
I have no symptoms to get tested.	407 (63.3%)	139 (21.6%)	97 (15.1%)
It is hard to get an appointment.	180 (28.0%)	253 (39.3%)	210 (32.7%)
Do you know the types of colon cancer screening available?	130 (20.2%)	513 (79.8%)	–
Barriers to colonoscopy as an early screening for colon cancer			
Colonoscopy is a painful procedure.	281 (43.7%)	275 (42.8%)	87 (13.5%)
Colonoscopy is expensive.	232 (36.1%)	292 (45.4%)	119 (18.5%)
Colonoscopy is embarrassing.	402 (62.5%)	174 (27.1%)	67 (10.4%)
It is difficult to get a colonoscopy appointment.	203 (31.6%)	304 (47.3%)	136 (21.2%)
Colonoscopy takes a lot of time.	251 (39.0%)	290 (45.1%)	102 (15.9%)
Colonoscopy is not important in my opinion.	72 (11.2%)	224 (34.8%)	347 (54.0%)
I am afraid of the results of my colonoscopy.	297 (46.2%)	198 (30.8%)	148 (23.0%)
I had a bad experience with a colonoscopy before.	62 (9.6%)	165 (25.7%)	416 (64.7%)
I do not know if I can have a colonoscopy.	260 (40.4%)	200 (31.1%)	183 (28.5%)
I do not have transportation to attend the colonoscopy.	111 (17.3%)	173 (26.9%)	359 (55.8%)
Barriers to FIT			
I think it is an unimportant test.	124 (19.3%)	184 (28.6%)	335 (52.1%)
I think it is an expensive test.	109 (17.0%)	275 (42.8%)	259 (40.3%)
I do not have time to do the test.	194 (30.2%)	203 (31.6%)	246 (38.3%)
Afraid of test results.	271 (42.1%)	187 (29.1%)	185 (28.8%)
I feel uncomfortable about how the test is done.	244 (37.9%)	195 (30.3%)	204 (31.7%)
I do not know where I can get the test.	325 (50.5%)	154 (24.0%)	164 (25.5%)

FIT = fecal immunochemical test.

Analysis of health care communication revealed a striking lack of physician-patient dialogue about CRC screening, with only 8.2% (n = 53) of participants having discussed screening with their health care providers, while the vast majority (n = 590, 91.8%) had never engaged in such discussions.

Cultural and social influences on colonoscopy decision were reported by approximately one-third of participants (n = 207, 32.2%), while 67.8% (n = 436) indicated no such influences. Accessibility emerged as a crucial factor, with 84.0% (n = 540) indicating they would be more likely to undergo screening if access was simplified.

There was overwhelming support for enhanced education initiatives, with 89.3% (n = 574) expressing the need for more education and awareness programs, while only 10.7% (n = 69) believed current educational efforts were sufficient.

Regarding comfort levels in discussing CRC screening with family and friends, responses were mixed. While 36.7% (n = 236) reported being very comfortable with such discussions and 29.7% (n = 191) were somewhat comfortable, a considerable proportion expressed discomfort (n = 111, 17.3%) or preferred not to disclose such information (n = 105, 16.3%). This distribution suggests varying levels of social stigma and personal comfort in addressing CRC screening within social networks (Table 4).

**Table 4:** Colon cancer education needs and comfort level in discussion.

Statement	N (%)
Have you ever discussed colon cancer screening with your doctor?	
Yes	53 (8.2%)
No	590 (91.8%)
Are there cultural or social factors that influence your decision to have a colonoscopy?	
Yes	207 (32.2%)
No	436 (67.8%)
You are more likely to get screened for colon cancer if it is easier to get.	
Yes	540 (84.0%)
No	103 (16.0%)
Do you think there is a need for more education and awareness programs to promote colon cancer screening?	
No, there is enough education and awareness.	69 (10.7%)
Yes, more programs are needed.	574 (89.3%)
How comfortable are you discussing colon cancer screening with your family and friends?	
Better not to disclose	105 (16.3%)
Somewhat comfortable	191 (29.7%)
Uncomfortable	111 (17.3%)
Very comfortable	236 (36.7%)

The age distribution of participants showed that 59.9% (n = 385) were under 45 years old, while 40.1% (n = 258) were aged 45 years or older. Among those over 45 years old, only 15.1% (n = 39) reported having undergone CRC screening, whereas 82.6% (n = 213) had not been screened, and 2.3% (n = 6) did not provide a response.

For participants who had not undergone screening, the most reported reason was lack of a doctor’s recommendation (56.4%, n = 119), followed by lack of awareness about the examination (46.9%, n = 99). Fear of examination was cited by 35.5% (n = 75) of respondents, while discomfort with the examination and embarrassment associated with the procedure were each reported by 22.7% (n = 48) of participants. Financial constraints were identified as a barrier by 12.3% (n = 26). Less frequently cited reasons included lack of symptoms (2.4%, n = 5), long waiting times (0.5%, n = 1), and being too busy (0.5%, n = 1).

Among those who had undergone screening, the primary motivating factor was a doctor’s recommendation (73.7%, n = 28). Family history of colon cancer was reported by 39.5% (n = 15) of screened participants, while awareness of screening importance was cited by 26.3% (n = 10). Other unspecified reasons accounted for 2.6% (n = 1).

**Figure 1.**
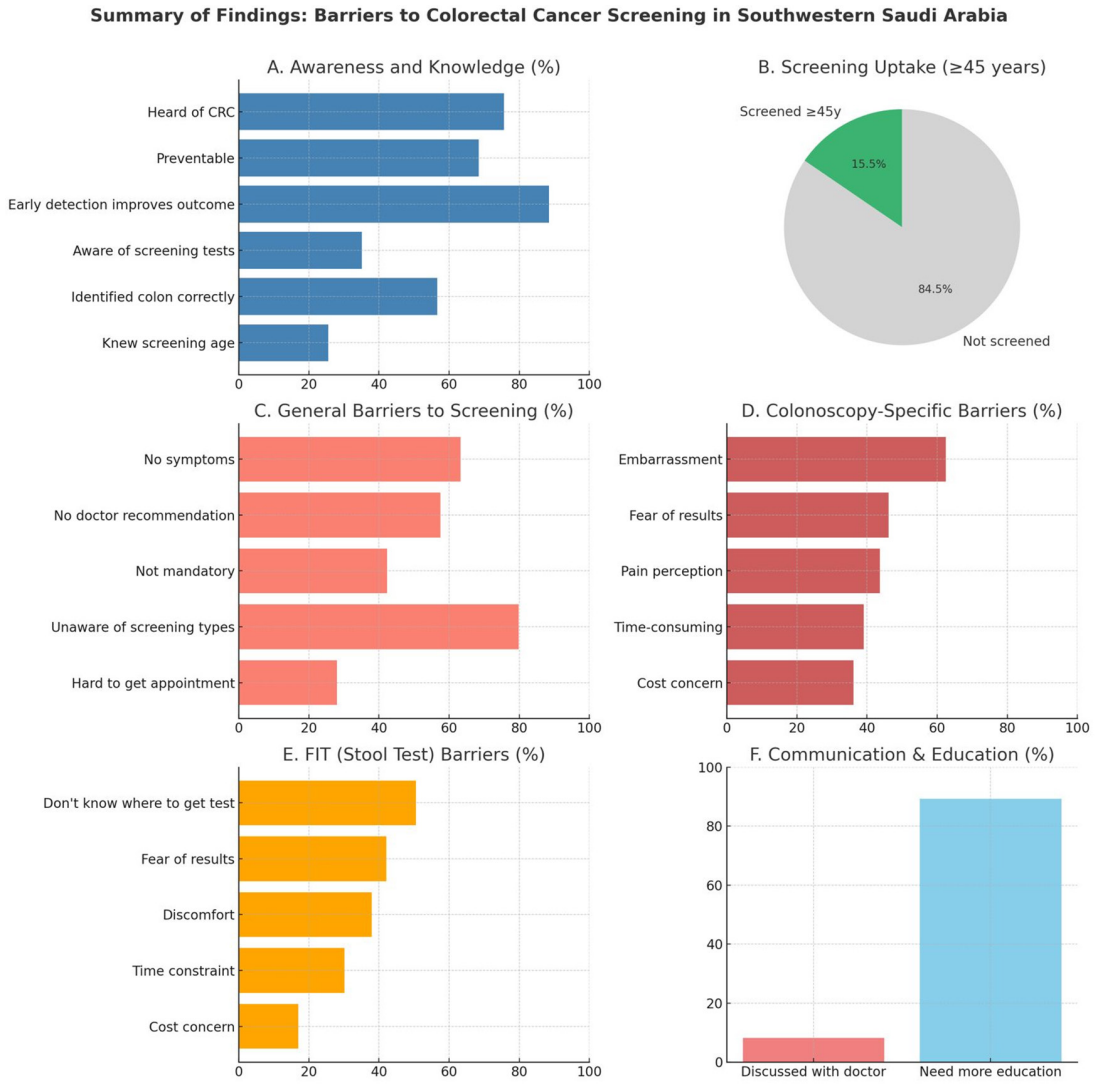
Barriers to CRC screening in southwestern Saudi Arabia. CRC = colorectal cancer.

When asked about overcoming barriers to CRC screening, most participants (83.7%, n = 538) suggested increasing public awareness campaigns. Providing more accessible and affordable screening options were supported by 73.3% (n = 471), improving communication between health care providers and patients by 64.2% (n = 413), and reducing screening costs by 62.5% (n = 402). Other suggestions, each mentioned by 0.2% (n = 1) of participants, included mandating screening, speeding up appointments, addressing the limitations of long-term appointments available only in the morning, and establishing specialized centers for CRC screening (Table 5). A graphic representation of the results has been constructed (Fig. 1).

**Table 5:** Colon cancer screening and barriers.

Variable	N (%)
Age	
<45	385 (59.9%)
>45	258 (40.1%)
If you are >45 years old, did you have a screening?	
Yes	39 (15.1%)
No	213 (82.6%)
Did not answer	6 (2.3%)
If No, what are the reasons?	
Lack of a doctor’s recommendation	119 (56.4%)
Lack of awareness of examination	99 (46.9%)
Fear of examination	75 (35.5%)
Discomfort with examination	48 (22.7%)
Embarrassment associated with examination	48 (22.7%)
Poor financial condition	26 (12.3%)
There are no symptoms	5 (2.4%)
Crowded appointments and long waits	1 (0.5%)
Too busy	1 (0.5%)
If Yes, what are the reasons?	
Awareness of the importance of screening	10 (26.3%)
Doctor’s recommendation	28 (73.7%)
Family history of colon cancer	15 (39.5%)
Others	1 (2.6%)
If Yes, when did you have the test?	
During the past 12 months	11 (28.2%)
1 year ago–3 years ago	16 (41.0%)
3–5 years ago	1 (2.6%)
5–10 years ago	6 (15.4%)
More than 10 years ago	5 (12.8%)
How can barriers be overcome?	
Increase public awareness campaigns	538 (83.7%)
Provide more accessible and affordable screening options	471 (73.3%)
Improving communication between health care providers and patients	413 (64.2%)
Reduce screening costs	402 (62.5%)
Mandate screening	1 (0.2%)
Speed-up appointments	1 (0.2%)
Long-term appointments and only available in the morning are the biggest barriers	1 (0.2%)
There are specialized centers for it	1 (0.2%)

## 4. Discussion

CRC is one of the leading causes of cancer-related morbidity and mortality worldwide.^[2]^ Most CRCs arise from polyp mutations over a long time.^[18,19]^ Therefore, screening for and removal of colonic polyps are associated with a decrease in the incidence of CRC.^[20–22]^ A study to evaluate the long-term risk of CRC and CRC-related mortality after colonoscopy showed that a negative high-quality colonoscopy was associated with a reduction in CRC and CRC-related mortality for up to 17 years.^[23]^ A systematic review showed that screening colonoscopy was associated with a 69% reduction in CRC incidence and decreased CRC-related mortality by 68%.^[24]^ Moreover, a case-control study among US veterans found that screening colonoscopy was associated with a 70% decrease in CRC mortality, including a 52% reduction in proximal CRC mortality.^[25]^ Another screening modality is the 2-step approach, where a noninvasive method (stool-based test) is utilized, and if positive, a colonoscopy is then performed. Large randomized controlled trials with long-term follow-up showed that biennial screening led to an 18% reduction in colorectal mortality,^[26,27]^ while annual fecal occult blood test screening was associated with 33% reduction in CRC over 30 years.^[28]^ Moreover, screening allows the detection of malignancy at an earlier stage, which is associated with better outcomes. CRC screening has been demonstrated to be cost-effective in Saudi Arabia.^[29]^ However, the benefit of screening depends on high participation rates, appropriate timing of referral, and therapeutic interventions for those who screen positive.^[5]^

Previous studies have investigated the knowledge and awareness of different age groups in different regions in Saudi Arabia. However, we wanted to investigate this in the southwestern region of Saudi Arabia, which has not been done before. Moreover, different studies studied different age groups including the general population, age 50 to 75, or age 40 and older.^[14–17,30–35]^ In our study, we targeted the general population for general knowledge, awareness, and perceived barriers and then for those who are at the age of screening were asked about their screening status and their perceived barriers towards colonoscopy-based or stool-based screening practices.

Our study showed that the majority of our study participants were aware of CRC and believed that early detection was associated with improved treatment outcomes (88%). However, only about a third (35%) were aware of cancer screening/detection tests for this disease. About 40% of the study participants did not know the anatomical location of the colon or incorrectly confused it with another gastrointestinal organ. Only about a quarter (25.5%) were able to correctly determine the appropriate age of screening, while, alarmingly, 17% thought that screening should be done only if there are symptoms. This high percentage signifies a significant gap in knowledge among the study population, which reflects the general population’s knowledge level.

When attempting to identify the most important barriers to CRC screening in general, about two-thirds reported that they do not screen because they do not have symptoms, and a similar percentage endorsed that lack of physician recommendation drives their decision not to screen. Around 79.8% of the participants were not aware of the available screening modalities.

Colonoscopy-specific barriers were studied due to the unique social and cultural characteristics of the Saudi population towards colonoscopy. Emotional and psychological barriers were responsible for most of the perceived barriers towards colonoscopy. Embarrassment was the most reported barrier (62.5%), followed by the fear of finding something sinister (46.2%), followed by concern about pain (40.4%). It is interesting that about a third of the participants were worried about financial barriers, although screening through governmental hospitals is free of charge.

We investigated the barriers to the implementation of FIT as a CRC screening, and we found that half of the study participants did not know about the test, and 42% were worried about the results, followed by a significant proportion (37.9%) being afraid of discomfort associated with the testing process. The significant proportion of participants being worried about pain associated with the fecal occult blood test underscores the importance of nationwide educational events.

Our study showed that physician recommendations for screening practices are still suboptimal, with only 8.2% having a discussion with their health care provider about screening. Around 89.3% of the study participants expressed the need for enhanced educational programs and screening awareness events.

The uptake of CRC screening was very low in our study population. Of those older than 45 years of age, only 15% of them reported having undergone CRC screening. Again, those who did not undergo screening reported that not being recommended by a physician and a lack of awareness about screening were reasons for not being screened. Of those who got screened, the primary motivator was a physician’s recommendation, which highlights the importance of physician recommendations in this population.

It is notable that most participants advocated for increased public awareness campaigns and improved dialogue between physicians and patients to overcome CRC screening barriers.

Our findings are similar to those of Alabdulkader et al, who found that only 10% of their participants had CRC screening education from their physician.^[16]^ Also, they found that only about 10% of those older than 40 years old underwent screening despite the majority being aware of CRC and its preventable nature. Another study that queried medical students found that fear of finding cancer was a major barrier to CRC screening and accounting for (56.7%), experiencing embarrassment (53.1%), and lack of awareness about the disease (52.7%).^[35]^ This fear of finding cancer on screening was also demonstrated in another study among participants from 2 tertiary hospitals, where nearly all participants were concerned that CRC screening would reveal cancer.^[36]^ Moreover, only 8.6% of participants from the eastern region of Saudi Arabia reported prior CRC screening in another study, with fear of results, embarrassment, and lack of time as major barriers.^[30]^ Interestingly, lack of physician recommendations was also cited as a major barrier to screening in this study population. A study involving teaching staff found that only 19% were aware of CRC screening, and only 23% were aware of the preventable nature of CRC.^[32]^ CRC screening has been proven to be cost-effective in Saudi Arabia.^[29,37]^ However, although the majority of participants had heard of CRC and its preventable nature, they were not aware of the screening methods and reported a lack of physician recommendations as a significant barrier to screening. This has been shown in our study and is in line with previous studies. To solve this, we should encourage primary care physicians to emphasize the importance of CRC screening to patients during their interactions. Moreover, increasing public awareness about CRC screening would mitigate the barrier of physician recommendation, which was also endorsed by Zacharakis and colleagues.^[29]^ We should note that screening participation requires time to reach an optimal uptake level, as it took Kaiser Permanente in Northern California 15 years to increase screening participation from 38.9% in 2000 to 82.7% in 2015 when they shifted from opportunistic screening to an organized direct-to-patient annual FIT outreach program.^[38]^

Our study has a good sample size of 643 participants, representing different age groups, including those above age 45 years. It assessed both knowledge about CRC, its screening modalities, and perceived barriers to CRC screening. This was done across different settings and targeting different educational and professional backgrounds. Generalizability is limited given that it represents participants from 2 cities within the southern region and should be interpreted in the context of a similar population. The use of convenience sampling and online distribution may have led to selection bias favoring younger, more educated participants who are able to use electronic and online resources. To counter this, we utilized in-person questionnaires in primary care clinics, malls, and common areas which offer in-person assistance to complete the questionnaire to the older population. Also, the participants were stratified according to age.

## 5. Conclusion

Despite fair awareness of risk factors and symptoms, there were significant knowledge gaps about screening procedures, with 79.8% of participants uninformed of available screening options. Among participants aged 45 and up, the screening rate was alarmingly low (15.1%), with major barriers indicated as a lack of physician recommendation/referral (53.1%) and knowledge gaps (46.9%). The fact that 91.8% of participants had never discussed CRC screening with their health care providers demonstrates an enormous communication gap. These findings highlight the critical need for focused educational programs, greater physician-patient communication, and increased accessibility to screening services.

## Author contributions

**Conceptualization:** Abdullah A. Al-Shahrani, Omair M. Alshahrani, Mohammed A. Ogran, Faisal M. AlShehry, Saifaleslam A. Mahmoud.

**Data curation:** Abdullah A. Al-Shahrani, Omair M. Alshahrani, Dima M. Alshahrani, Mohammed A. Ogran, Faisal M. AlShehry, Saifaleslam A. Mahmoud, Yara A. Alshehri.

**Formal analysis:** Abdullah A. Al-Shahrani, Ibrahim H. Tawhari.

**Methodology:** Abdullah A. Al-Shahrani.

**Project administration:** Abdullah A. Al-Shahrani.

**Supervision:** Abdullah A. Al-Shahrani.

**Writing – original draft:** Abdullah A. Al-Shahrani.

**Writing – review & editing:** Abdullah A. Al-Shahrani, Omair M. Alshahrani, Dima M. Alshahrani, Mohammed A. Ogran, Faisal M. AlShehry, Saifaleslam A. Mahmoud, Yara A. Alshehri, Yahya Shabi, Abdulrahman Alloghbi, Ibrahim H. Tawhari, Mohammed A. Alzahrani.


