# Supplementary material for: Colorectal cancer screening challenges in southwestern Saudi Arabia
Source: Medicine (Baltimore). 2026 Jun 12;105(24):e49289. doi: 10.1097/MD.0000000000049289 (PMC13268442; doi:10.1097/MD.0000000000049289)
Supplement: Supplementary file 1 [file medi-105-e49289-s001.docx]

***Table 1. Participants’ knowledge about colorectal cancer***

| **Statement** | **Yes** | **No** | **I Don't Know** |
| --- | --- | --- | --- |
| **General knowledge about Colon Cancer and Screening** | | | |
| Have you heard of colon and rectal cancer before? | 487 (75.7%) | 156 (24.3%) | - |
| What is your first impression when you hear about colon cancer? | 116 (18.0%) | 418 (65.0%) | 109 (17.0%) |
| Do you think colon cancer is common in Saudi Arabia? | 319 (49.6%) | 94 (14.6%) | 230 (35.8%) |
| Do you think it is possible to treat colon cancer when detected early? | 569 (88.5%) | 18 (2.8%) | 56 (8.7%) |
| Is Colon Cancer a Preventable Disease? | 440 (68.4%) | 40 (6.2%) | 163 (25.3%) |
| Have you ever heard of any test or examination used to detect colon cancer? | 226 (35.1%) | 318 (49.5%) | 99 (15.4%) |
| Do you think colon cancer is not a serious health threat? | 148 (23.0%) | 384 (59.7%) | 111 (17.3%) |
| Do you think that screening for colon cancer is useless? | 107 (16.6%) | 433 (67.3%) | 103 (16.0%) |
| **Risk Factor** | | | |
| Age | 344 (53.5%) | 150 (23.3%) | 149 (23.2%) |
| Inactive (sedentary) lifestyle | 430 (66.9%) | 89 (13.8%) | 124 (19.3%) |
| Obesity | 402 (62.5%) | 113 (17.6%) | 128 (19.9%) |
| Eating red meat and food low in fiber | 380 (59.1%) | 110 (17.1%) | 153 (23.8%) |
| Smoking | 464 (72.2%) | 71 (11.0%) | 108 (16.8%) |
| Inflammatory bowel diseases (Crohn’s, ulcerative colitis) | 459 (71.4%) | 31 (4.8%) | 153 (23.8%) |
| Colon polyps | 402 (62.5%) | 49 (7.6%) | 192 (29.9%) |
| Family history (colon cancer in first-degree relatives) | 471 (73.3%) | 65 (10.1%) | 107 (16.6%) |
| Genetic factors | 454 (70.6%) | 65 (10.1%) | 124 (19.3%) |
| **Symptom** | | | |
| Abdominal pain | 393 (61.1%) | 100 (15.6%) | 150 (23.3%) |
| Unintentional weight loss | 470 (73.1%) | 45 (7.0%) | 128 (19.9%) |
| Sudden change in bowel habits (diarrhea or constipation) in the elderly | 445 (69.2%) | 41 (6.4%) | 157 (24.4%) |
| Blood in stool or dark stool | 447 (69.5%) | 31 (4.8%) | 165 (25.7%) |
